# ROS and Oxidative Stress in Stem Cells

**DOI:** 10.1155/2017/5047168

**Published:** 2017-09-06

**Authors:** Artur Cieślar-Pobuda, Jianbo Yue, Hsin-Chen Lee, Magdalena Skonieczna, Yau-Huei Wei

**Affiliations:** ^1^Stem Cell Group, Nordic EMBL Partnership, Centre for Molecular Medicine Norway (NCMM), University of Oslo, Oslo, Norway; ^2^City University of Hong Kong, Kowloon, Hong Kong; ^3^Center for Mitochondrial Medicine and Free Radical Research, Changhua Christian Hospital, Changhua City, Taiwan; ^4^Biotechnology Centre, Silesian University of Technology, Gliwice, Poland; ^5^Mackay Medical College, New Taipei City, Taiwan

Reactive oxygen species (ROS) are well known to be implicated in various important cellular processes including signaling, regulation of homeostasis, or induction of death. Oxidative stress resulting from increased ROS production and impaired free radical scavenging systems can cause severe damage to biological macromolecules, affecting cell proliferation and causing genomic instability and cellular senescence. Although ROS are involved in a wide range of cellular processes, a limited number of studies have examined the generation and function of ROS in stem cells. It is known that ROS may enhance differentiation of stem cells and facilitate reprogramming into induced pluripotent stem cells (iPSCs), but on the other hand, they are also associated with malignant transformation or premature aging. Stem cells have also been shown to possess defective DNA repair machinery, which may have serious consequences for cells exposed to extensive oxidative stress. Since stem cells are considered as a promising tool in regenerative medicine, it is crucial to know and better understand all the processes related to ROS to prevent potential generation of mutations causing genomic instability and to avoid unwanted ROS-driven differentiation. Thus, the mechanisms by which genomic integrity of stem cells is maintained under oxidative stress and the role of ROS should be elucidated before stem cells finally find their place in clinical applications.

The paper by C.-J. Li et al. demonstrates that treatment with antioxidants not only increases proliferation and mitochondrial integrity of human mesenchymal stem cells (hMSCs) but also enhances the therapeutic potential of hMSCs by promoting the formation of tunneling nanotubes (TNTs) between hMSCs and oxidatively injured cells in a coculture system. The authors demonstrate that TNTs enable hMSCs to “export” their healthy mitochondria to the injured cells and hence decrease their oxidative stress and stabilize the mitochondrial membrane potential of the injured cells. Concurrently, the rescued cells also show enhanced mitophagy, implicating that their damaged mitochondria are eliminated in order to maintain normal cell physiology. The findings highlight that antioxidants enhance mitochondrial transfer from hMSCs to the injured cells and provide them with a repair mechanism.

A similar topic is presented by Y.-C. Chuang et al. who show that Wharton's jelly mesenchymal stem cells (WJMSCs) are able to transfer healthy mitochondria to cybrid cells from a patient with myoclonus epilepsy associated with ragged-red fibers (MERRF) through intercellular connections. This mitochondrial transfer is found to be associated with reduction of oxidative stress and improvement of mitochondrial bioenergetics in MERRF cybrid cells. The ability of WJMSCs to “help” cells with defective mitochondrial function (e.g., MERRF cells) through donating healthy mitochondria is an intriguing phenomenon that may provide new cues for the development of more effective treatment of diseases caused by or associated with mitochondrial dysfunction.

V. A. Sergeeva et al. contributed an interesting study, in which the adaptive response of MSCs to low doses of ionizing radiation (IR) is described. The authors show that such a response may be mediated by oxidized cell-free DNA (cfDNA) fragments. It is demonstrated that treatment of MSCs with low doses of IR leads to cell death of part of the cell population and release of oxidized cfDNA, which has the ability to penetrate into the cytoplasm of other cells. Oxidized cfDNA, like low doses of IR, induces ROS production, ROS-induced oxidative DNA damage, cell cycle arrest, activation of DNA repair mechanisms, and inhibition of apoptosis. The MSCs pretreated with a low dose of irradiation or oxidized cfDNA are equally effective in induction of an adaptive response to the challenge of further doses of radiation. This study suggests that oxidized cfDNA is a stress-triggered signaling molecule that mediates radiation-induced bystander effects and that it is an important component of radioadaptive responses of cells to low doses of IR.

D. Sainz de la Maza et al. explore the role of ROS and autophagy on reprogramming of primordial germ cells (PGCs) into pluripotent cells. The authors demonstrate that a metabolic shift from oxidative phosphorylation toward glycolysis, autophagy, and mitochondrial inactivation and an early rise in ROS levels are necessary for PGC reprogramming. It is shown that all these processes are regulated by a correct (right) HIF1/HIF2 balance and Oct4. The cells obtained are unable to self-renew, and it is postulated that *Blimp1* may be responsible for this.

J. Kučera et al. focus in their work on the effect of hypoxia on intracellular signaling pathways responsible for mouse embryonic stem (ES) cell maintenance. By employing wild-type and hypoxia inducible factor 1- (HIF-1-) deficient ES cells and measuring phosphorylation of proteins of the ERK, Akt, and STAT3 pathways, the authors investigate the response of ES cells to hypoxia. The study shows that ERK signaling is downregulated in hypoxia in a ROS-dependent manner, but without the involvement of hypoxia-inducible factor HIF-1. The authors also observe a decreased ROS level in hypoxia and a similar phosphorylation pattern for ERK when the cells are supplemented with glutathione.

Finally, the review article by M. Skonieczna et al. gives an extensive overview about NADPH oxidases (NOX) and their role in various cellular systems. The article describes the functions and mechanisms of action of NOX and NOX-derived ROS in stem cells, cancer cells, and cancer stem cells. It also points out the importance of understanding NOX-dependent cellular processes, as a future perspective for regenerative medicine and development of new therapies toward cancers.

In conclusion, the aim of this special issue is to provide updated information for readers to better understand the role of oxidative stress and ROS in stem cell physiology. All the articles selected for this special issue present important findings that will undoubtedly provide useful cues for future research.

*Artur Cieślar-Pobuda* *Jianbo Yue* *Hsin-Chen Lee* *Magdalena Skonieczna* *Yau-Huei Wei*

